# Evaluation of Unsolicited Feedback from Patients with Cancer and Their Families as a Strategy to Improve Cancer Care Delivery

**DOI:** 10.3390/curroncol31050186

**Published:** 2024-04-28

**Authors:** Parvaneh Fallah, Lucas Clemons, Michelle Bradbury, Lisa Vandermeer, Mark Clemons, Julie Renaud, Marie-France Savard

**Affiliations:** 1 Division of Medical Oncology and Department of Medicine, Ottawa Hospital Cancer Centre and University of Ottawa, Ottawa, ON K1N 6N5, Canada; parvaneh.fallah@mail.mcgill.ca (P.F.); michelle.bradbury@medportal.ca (M.B.); mclemons@toh.ca (M.C.); 2 Ottawa Hospital Research Institute, Ottawa, ON K1Y 4E9, Canada; lucas.clemons@mail.mcgill.ca (L.C.); lvandermeer@ohri.ca (L.V.); 3 Ottawa Hospital Cancer Centre, Ottawa, ON K1H 8L6, Canada; jurenaud@toh.ca

**Keywords:** unsolicited feedback, cancer care delivery, patient experience, complaints, compliments

## Abstract

Background: Unsolicited patient feedback (compliments and complaints) should allow the healthcare system to address and improve individual and overall patient, family, and staff experiences. We evaluated feedback at a tertiary cancer centre to identify potential areas for optimizing care delivery. Methods: unsolicited feedback submitted to the Patient Relations Department, relating to the Divisions of Medical and Radiation Oncology, at the Ottawa Hospital, was analyzed. Results: Of 580 individual reports submitted from 2016 to 2022, patient demographics were available for 97% (563/580). Median patient age was 65 years (range 17–101), and 53% (301/563) were female. The most common cancer types were breast (127/545, 23%) and gastrointestinal (119/545, 22%) malignancies, and most (64%, 311/486) patients had metastatic disease. Feedback was submitted mainly by patients (291/579, 50%), and predominantly negative (489/569, 86%). The main reasons for complaints included: communication (29%, 162/566) and attitude/conduct of care (28%, 159/566). While feedback rates were initially stable, an increase occurred from 2019 to 2021. Conclusions: Unsolicited feedback remains mostly negative, and relates to physician communication. If we are to drive meaningful changes in care delivery, more standardized means of assessing feedback and implementation strategies are needed. In addition, in an era of increased healthcare provider burnout, strategies to enhance formal positive feedback are also warranted.

## 1. Introduction

Feedback from patients with cancer and their families can help healthcare providers understand how patients experience healthcare services, and hopefully improve them [1,2]. While feedback can either be solicited or unsolicited, it can be broadly defined as ‘an individual patient or family visitor’s subjective perspective on medical services received’ [3]. The literature suggests that unsolicited patient complaints about physician practices are associated with increased malpractice claims and reduced clinical quality [4]. In the cancer care setting, unsolicited complaints are more commonly about medical oncologists than radiation oncologists, and are also more common in recent graduates and those working at academic centers [4]. Most complaints relate to the categories of ‘patient-provider relationships’, ‘humanness and caring’, ‘communication’ and ‘patient–staff dialogue’ [5].

The Ottawa Hospital Cancer Centre is a large tertiary cancer centre providing cancer services for a population off 1.2 million. Within the centre, the Divisions of Medical Oncology and Radiation Oncology see approximately 5 to 6 thousand new consultations each, and 60 and 24 thousand follow up visits per year, respectively. Unsolicited patient-related feedback (both compliments and complaints) for all disciplines is processed through a specific Patient Relations Department. This team consists of professionals from various health care backgrounds who possess a wide range of skills and expertise, with the goal of collecting and addressing patient inquiries, feedback, and concerns.

Given that there is uncertainty as to whether patient feedback actually leads to improvements in services as a whole, we decided to evaluate the nature of unsolicited compliments and complaints to determine whether there was the possibility of systemic changes from these reports that could actually improve patient, family, and staff satisfaction. The hospital has undergone several major service modifications in recent years (including a fundamental change in the nursing model in ambulatory care, new electronic medical record [EMR], patient online access to EMR, COVID pandemic-associated measures, and the increased use of Virtual Care) that have profoundly changed the way care is delivered. Because of these changes, we were also interested in reviewing and comparing formal patient feedback over this period. Finally, as there appears to be very little in the literature with respect to positive feedback, in an era of increased healthcare provider burnout, we also wanted to assess whether actionable patterns for this type of feedback exist.

## 2. Materials and Methods

### 2.1. Study Population and Data Source

After approval by the Ottawa Health Science Network Research Ethics Board (OHSN-REB 2022-0462), we obtained a dataset from the Ottawa Hospital Patient Relations Department containing summary data of all patient feedback that was submitted between April 2016 and April 2022 relating to care delivered by the Divisions of Medical and Radiation Oncology. According to the Patient Relations Department, unsolicited patient-related feedback is defined as explicit communication by telephone, email, or regular mail from a patient or their family about their experience with any member of staff (e.g., nursing, physicians, clerks, and other hospital staff) to the Patient Relations Department.

April 2016 was chosen as the starting point, given the major changes in how care is delivered occurred after this time, including: the hospital oncology transformation project (which included the move from a patient-designated nurse model to a tumor site-based model, 2015–2019), the implementation of a new EMR system (EPIC, June 2019), COVID-19 restrictions with increased use of virtual care (March 2020 to 2022), and making patient medical records, as well as imaging reports, virtually accessible (MyChartTM, 2017; imaging reports, 2021).

### 2.2. Data Collection

Data was retrospectively extracted from the dataset provided by the Patient Relations Department, and divided into four broad categories.

Who (e.g., patient, spouse, children, others) communicated with the Patient Relations Department.

The service to which the feedback was directed (e.g., medical oncology, radiation oncology, cancer program including registration office, patient support line, weekend care path, other), and the discipline of the staff to whom the feedback was directed (i.e., physician, nursing, booking clerk, other).

The nature of the feedback (i.e., positive, negative, n/a) and the reason for feedback. These reasons were broadly grouped into: attitude and conduct (e.g., patient was admitted to hospital and was not treated well by the team), professional skills (e.g., no physical examination performed during the visit, or the patient wanted to change physicians as they thought the doctor was not providing appropriate care), wait times (e.g., booking appointment, punctuality, radiology/imaging, biopsy, other tests, insurance forms, drug reimbursement, prescription renewals, treatment/surgery, pathology report/test results, admission/beds), communication (e.g., listening, explaining, sharing information within circles of care, communicating test results/appointment time or date), patient expectations (e.g., hospital room was available when the patient arrived, language of choice, virtual care, MyChart).

The resolution as perceived by the Patient Relations Department (i.e., achieved, not achieved, not mentioned, not applicable).

Data collection was performed by one reviewer (PF), and 25% of the data was randomly sampled by a second reviewer (MFS) to ensure the accuracy of the collected information.

### 2.3. Demographic Data

Provided the patient gave permission at the time of their unsolicited feedback, the feedback was linked to the patient’s chart number. The patient’s chart number was used to collect de-identified patient demographics (i.e., age, gender, ethnicity, type and stage of cancer) from their EMR. To preserve patient confidentiality and privacy with respect to the complaint, this was performed by a third reviewer (MB) who did not have access to any of the feedback details.

### 2.4. Data Analysis

Data was reported using descriptive statistics, primarily measures of frequency, and analyzed using Microsoft Excel.

## 3. Results

### 3.1. Baseline Patient and Disease Characteristics

Of 580 individual reports submitted between April 2016 and April 2022, 52 occurred in 2016, 60 in 2017, 74 in 2018, 107 in 2019, 120 in 2020, 121 in 2021, and 45 in 2022 (Table 1).

**Figure 1:**
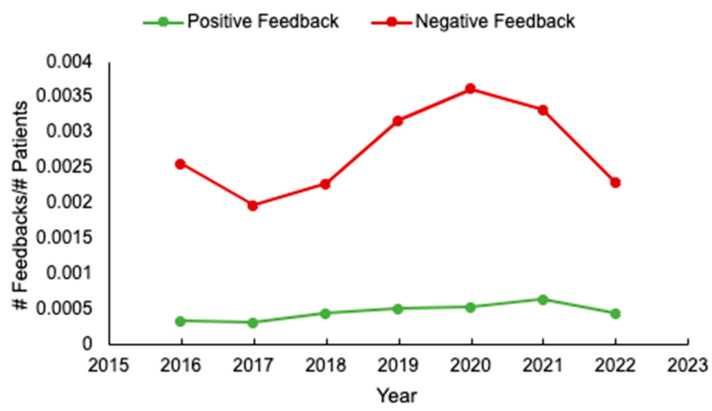
The proportion of positive and negative feedback adjusted per patient evaluated by year.

When adjusting for the number of patients seen during this time period, there was a rise in the number of complaints from 2019 to 2021, while compliments remained stable (Figure 1). The numbers of complaints for the first four months of 2022 fell closer to the 2016–2018 levels.

Most feedback was submitted by either the patient (50%, 291/579), their children (20%, 116/579), or their spouse (16%, 94/579) (Table 1). Patient demographics were available for 563 (97%) of reports (Table 1). The median patient age was 65 years (range 17 to 101), and 53% (301/563) were female. Among the reported ethnicities, 70 results were available, and the most common were Caucasian (79%), Asian (10%), and Middle Eastern (6%). The most common cancer types were breast (22.6%, 127/562), gastrointestinal (21.2%, 119/562), lung (13.7%, 77/562), and genitourinary (12.5%, 70/562). Patients had metastatic disease in 64% (311/486) of cases. Overall, when evaluated by both tumour site and stage, the most common sources of feedback were stage 4 gastrointestinal cancers (87/562, 15%) and stage 1–3 breast cancer (75/562, 13%).

### 3.2. Services and Staff to Which the Feedback Was Directed

Feedback was directed at the medical oncology division (34%, 117/348), the cancer program (30%, 106/348), and the radiation oncology division (21%, 72/348) (Table 2). Only 1% (4/348) of feedback was addressed to multiple divisions. The specific staff named in the feedback were physicians (67%, 282/423), nurses (22%, 95/423), booking clerks (11%, 47/423), and other staff (15%, 62/423) in the oncology division. Feedback identified multiple different staff in 16% (63/423) of cases.

### 3.3. Nature of the Feedback

Most unsolicited feedback was negative (86%, 489/569). With respect to positive feedback, the radiation oncology division (18%, 13/72) and the nursing staff (51%, 48/95) received the highest proportion of unsolicited positive feedback. Specific reasons for feedback are outlined below, and the three most common categories of feedback by year are presented in Supplemental Materials Figure S1. Proportion of feedback per division and per type of staff is presented in Supplemental Materials Figures S2 and S3.

#### 3.3.1. Reasons for Complaints

With respect to the reasons for complaints, the most common reasons were: communication (38%, 158/489), attitude and conduct of care (17%, 82/489), patient expectation (17%, 84/489), waiting time (15%, 74/489), and professional skills (11%, 53/489). The feedback related to the COVID-19 pandemic restrictions reached 8% (44/489). Over 60 feedback reports provided included more than one of the above reasons.

The communication and wait time themes were further subdivided to better understand the nature of the complaints. For “communication” (158 complaints), there were complaints with regard to: explaining (47%, 75/158), sharing information (22%, 35/158), not reaching patient support line/care, path/doctor, office/psycho-social, team/booking clerk (18%, 29/158), communicating test results, appointment time, or date (8%, 12/158), and listening (3%, 5/158). For “wait times”, the main complaints were regarding: appointment booking (28%, 21/74), delay in treatment/surgery (28%, 21/74), punctuality (11%, 8/74), and drug reimbursement (9%, 7/74) (Table 2 and Supplementary Table S1).

#### 3.3.2. Reasons for Compliments

While feedback was predominantly negative, 82 out of 469 responses were positive (14%). Among the positive feedback, 89% (73/82) involved attitude and conduct, 7% (6/82) professional skills, 4% (3/82) communication, 2% (2/82) patient expectation, and 1% (1/82) MyChart.

### 3.4. Resolution

The Patient Relations Department collects data on whether complaints were resolved. In total, 89% (298/336) of these complaints were resolved.

## 4. Discussion

To provide high-quality and person-centered care, it is important to understand the patient and their families’ experience with the care they received [6]. As patient experience has been positively associated with clinical safety and effectiveness outcomes, it is now accepted as an important indicator to monitor and improve service standards [2,7]. In recent years, there have been several changes in the way care is delivered at The Ottawa Hospital Cancer Centre, with the aim of optimizing the patient experience and improving effectiveness in care delivery. In this study, we analyzed retrospective unsolicited feedback communicated to The Ottawa Hospital through the Patient Relations Department to better understand any changes in patients’ satisfaction or concerns with regard to the change in care delivery, as well as to identify possible proactive strategies to improve both the patient and staff experience and satisfaction.

We observed that the amount of negative feedback almost doubled in years 2019, 2020, and 2021, as compared to the 2016 to 2018 period. During the 2019–2021 period, many important changes in cancer care delivery could explain the increase in complaints, including: the move from a patient-designated nurse model (nurse assigned to patient) to a tumor site-based (nurse assigned to a tumor site, not to a specific patient) model (2018–2019), the implementation of a new electronic medical record system (EPIC, June 2019), COVID-19 restrictions with rapid adoption of virtual care (March 2020–2022) and making patient imaging reports virtually accessible to the patient (2021). Unfortunately, we are unable to assess the individual impact of all these changes.

The most common feedback themes observed were communication and attitude and conduct. Poor communication was flagged in other studies as a major complaint reason in Canada and other countries [5,8]. In the literature, it was reported that newer EMRs, such as EPIC, caused healthcare workers to spend more time on chart reviews, documentation, and ordering functions [9,10]. As a result, less time is spent with patients and caregivers, possibly contributing to patient dissatisfaction [11]. Furthermore, one could hypothesize that having access to imaging reports would improve communication and possibly reduce negative feedback, but this was not observed in our study. While communication remains the top issue from the 2019 to 2021 period, feedback related to the COVID-19 pandemic was observed only for 2020 (Supplemental Materials Figure S1). Therefore, despite our expectations, the COVID-19 pandemic is not the main cause for the rise in patient complaints between 2019 and 2021. This has been reported elsewhere, when the COVID-19 pandemic and its restrictions were not associated with more patient complaints in an Irish hospital [12].

Patients with breast cancer had the most negative feedback reported among different cancer sites. While breast cancer is the most prevalent cancer type in women, it does not explain the significantly higher rate of feedback reports per patient when compared to genitourinary cancers (which include prostate cancer, the highest prevalent cancer in men) and gastrointestinal cancers (this disease site includes patients with colorectal, hepatobiliary pancreatic, gastric, and esophageal cancers) [13]. Based on this, it may be advisable to implement targeted strategies to address the higher rate of feedback from breast cancer patients.

There are several limitations to this study. First, it was a single-centre study, and related only to the Divisions of Medical and Radiation Oncology, which limits the generalizability of the findings. It would be interesting to evaluate the patient and family feedback in other centres where EMRs (e.g., EPIC) and other changes in healthcare delivery have been newly implemented. Furthermore, the Department of Patient Relations does not have a unified tool to prospectively obtain patients’ feedback in a standardized way or, indeed, to assess their evaluation of whether resolution had occurred. Positive feedback is often given by patients directly to the health care provider by various means (e.g., verbal, appreciation card), and may not have been captured in the Patient Relations Department database. On the other hand, negative feedback is more likely to be reported to this department, as it is anonymous and, thus, safer than complaining directly to staff members for fear of having care negatively impacted. Finally, some important details regarding the feedback were not accessible or recorded, including: physician demographics (gender and age), patient ethnicity, how negative feedback was resolved, and how this may have affected patient care.

Unsolicited feedback is a source of opportunity and challenges. Despite the importance of patient feedback in improving patient care, no concerted way was found of using this information to improve our local healthcare delivery. This is likely for a number of reasons, including: the relatively small number of patients receiving care that make complaints, as well as a lack of standard approach to assess complaint severity, analyze it, and catalogue the actions taken in response to complaints [14,15]. As most of the unsolicited feedback was negative, and from a relatively small number of patients, one could question how representative this feedback really is: are these experiences shared by a majority, and is action warranted in every case? One solution would be to encourage or trigger feedback on different themes raised by the unsolicited feedback to be able to better evaluate what is performed well and what is not. Positive feedback would also be essential in this context, and could represent a source of learning. Burnout among healthcare workers, especially physicians, and limited health care resources are major issues that need to be considered when addressing feedback [16,17,18]. The way feedback is handled will be critical in effecting health care improvement, as negative feedback is both a risk factor for increased burnout as well as an indicator for it. Future studies could incorporate this to develop a systematic and standardized approach to assess and implement feedback into practice.

## 5. Conclusions

Unsolicited feedback by patients are mainly complaints and related to physician communication. An increase in negative feedback by patients was observed from 2019 to 2021 in our centre, and multiple possible causes were identified. Counterintuitively, the COVID-19 pandemic only played a minor role in explaining the increase in patient complaints. Larger prospective studies involving multiple cancer centres with a unified approach to handle patient feedback, unsolicited and solicited, are needed to improve the healthcare system and ensure patient and healthcare provider needs are met.

## Figures and Tables

**Table 1:** Baseline patient and disease characteristics.

Characteristics	N	Total
**Age, Mean, (sd)**	563	63.7 (14.0)
Median age (range)		65 (17, 101)
<35 No. (%)		23 (4%)
36–50 No. (%)		60 (11%)
51–74 No. (%)		358 (64%)
75 < No. (%)		122 (22%)
Sex:	563	
Male, No. (% male)		262 (47%)
Female, No. (% female)		301 (53%)
Ethnicity:	70	
Asian (%)		7 (10%)
Caucasian (%)		55 (79%)
Middle Eastern (%)		4 (6%)
Other (%)		4 (6%)
Cancer Type:	562	
Breast (%)		127 (22.6%)
- Stage 1–3 (%)		- 75 (59%)
- Stage 4 (%)		- 47 (37%)
- N/A (%)		- 5 (4%)
Central nervous system (%)		20 (3.6%)
Unknown primary (%)		4 (0.7)
Gastrointestinal (%)		119 (21.2%)
- Stage 1–3 (%)		- 30 (25%)
- Stage 4 (%)		- 87 (73%)
- N/A (%)		- 2 (2%)
Genitourinary (%)		70 (12.5%)
- Stage 1–3 (%)		- 23 (33%)
- Stage 4 (%)		- 47 (67%)
Gynecological (%)		24 (4.3%)
Head and Neck (%)		31 (5.5%)
Hematological (%)		33 (5.9%)
Lung (%)		77 (13.7%)
Sarcoma (%)		7 (1.2%)
Skin (%)		27 (4.8%)
Multiple sites ^1^ (%)		3 (0.5%)
Other (%)		3 (0.5%)
None (%)		17 (3.0%)
Overall Stage:	486	
1–3 (%)		175 (36%)
4 (%)		311 (64%)
Person Giving Feedback:	579	
Patient (%)		291 (50%)
Spouse (%)		94 (16%)
Children (%)		116 (20%)
Other (%)		78 (13%)

^1^ Multiple sites includes breast/lung, CNS/GU, and GI/lung.

**Table 2:** Feedback characteristics.

Feedback	N *	Total	Positive	Negative	N/A
Type of feedback	569 (+2)		82 (14%)	489 (86%)	
Division feedback addressed to:	348 (+4)				
Medical Oncology (%)		117 (34%)	10 (9%)	105 (90%)	2 (2%)
Radiation Oncology (%)		72 (21%)	13 (18%)	57 (79%)	2 (3%)
Cancer Program (%)		106 (30%)	16 (15%)	89 (84%)	1 (1%)
Other (%)		57 (16%) + 1	16 (28%)	41 (72%)	1 (2%)
Staff feedback addressed to:	423 (+63)				
Physician		282 (67%)	56 (20%)	222 (79%)	4 (1%)
Nursing		95 (22%)	48 (51%)	45 (47%)	2 (2%)
Booking Clerk		47 (11%)	2 (4%)	45 (96%)	0 (0%)
Other		62 (15%)	12 (19%)	46 (74%)	4 (1%)
Reason for Feedback:	566 (+70)				
Attitude and Conduct		159 (28%) + 1	74 (47%)	82 (52%)	4 (3%)
Professional Skills		59 (10%) + 2	7 (12%)	53 (90%)	1 (2%)
Waiting Time		74 (13%)	0 (0%)	74 (100%)	0 (0%)
Communication		162 (29%)	3 (2%)	158 (98%)	1 (1%)
Patients Expectation		86 (15%) + 1	2 (2%)	84 (98%)	1 (1%)
Language of Choice		2 (4%)	0 (0%)	2 (100%)	0 (0%)
Virtual Care		4 (1%)	0 (0%)	4 (100%)	0 (0%)
MyChart		15 (3%) + 1	1 (7%)	15 (100%)	0 (0%)
COVID Restriction		46 (8%)	0 (0%)	44 (96%)	2 (4%)
Other		29 (5%)	0 (0%)	29 (100%)	0 (0%)
If communication:	162				
Listening		5 (3%)	0 (0%)	5 (100%)	0 (0%)
Explaining		75 (47%)	0 (0%)	75 (100%)	0 (0%)
Sharing Information		35 (22%)	0 (0%)	35 (100%)	0 (0%)
Communicating test results/appointment time or date		11 (8%)	0 (0%)	11 (92%)	1 (8%)
Not reaching patient support line/care path/doctor office/psycho-social team/booking clerk		28 (18%)	0 (0%)	28 (100%)	0 (0%)
N/A		7 (4%)	3 (43%)	4 (57%)	0 (0%)
Resolution Achieved:	336				
Yes		298 (89%)			
No		38 (11%)			

* In the N column, data including a “+ number” (e.g., Type of feedback: 569 [+2]) represents cases in which respondents provided multiple complaints and/or compliments in a single feedback report.

## Data Availability

Data is available upon request and approval by the Ottawa Hospital Science Network Research Ethics Board.

## Supplementary Materials

The following supporting information can be downloaded at: https://www.mdpi.com/article/10.3390/curroncol31050186/s1. Figure S1: The most common feedback categories adjusted for patients seen by year; Figure S2: Proportion of feedback per division adjusted for patients seen by year; Figure S3: Proportion of feedback per type of staff adjusted for patients seen by year; Table S1: Characteristics of feedback related to waiting time.
