# Supplementary material for: Evaluation of Unsolicited Feedback from Patients with Cancer and Their Families as a Strategy to Improve Cancer Care Delivery
Source: Curr Oncol. 2024 Apr 28;31(5):2488–96. doi: 10.3390/curroncol31050186 (PMC11120482; doi:10.3390/curroncol31050186)
Supplement: Supplementary file 1 [file curroncol-31-00186-s001.zip › curroncol-2969628-supplementary.pdf]

Supplemental Material

Figure S1. The most common feedback categories adjusted for patients seen by year

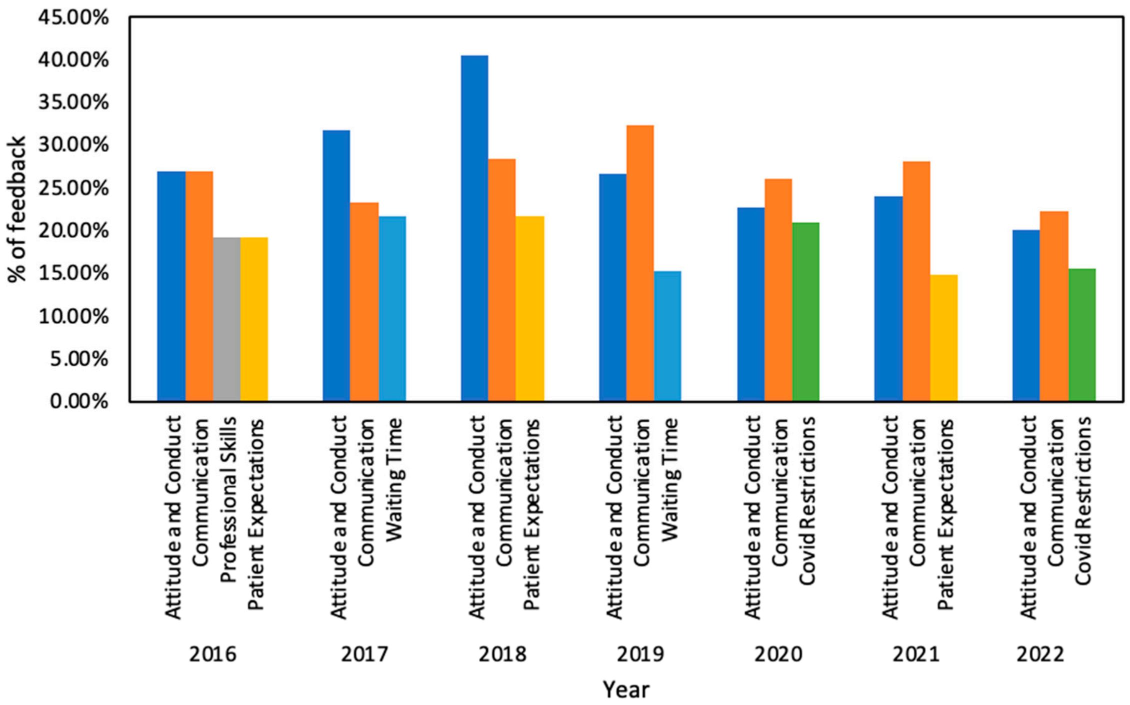

Table S1 Characteristics of feedback related to waiting time.

	N	Total	Positive	Negative	N/A
**Waiting time;**	74 (+1)				
Getting appointment:		21 (28%)	0 (0%)	21 (100%)	0 (0%)
Punctuality:		8 (11%)	0 (0%)	8 (100%)	0 (0%)
Radiology:		2 (3%)	0 (0%)	2 (100%)	0 (0%)
Biopsy to be done:		1 (1%)	0 (0%)	1 (100%)	0 (0%)
Other tests to be done:		4 (5%)	0 (0%)	4 (100%)	0 (0%)
Insurance/disability form:		2 (3%)	0 (0%)	2 (100%)	0 (0%)
Drug reimbursement:		7 (9%)	0 (0%)	7 (100%)	0 (0%)
Prescription renewal:		2 (3%)	0 (0%)	2 (100%)	0 (0%)
Delay in treatment/Surgery:		21 (28%)	0 (0%)	21 (100%)	0 (0%)
Delay in pathology report/test results:		4 (5%)	0 (0%)	4 (100%)	0 (0%)
N/A:		3 (4%)	0 (0%)	3 (100%)	0 (0%)

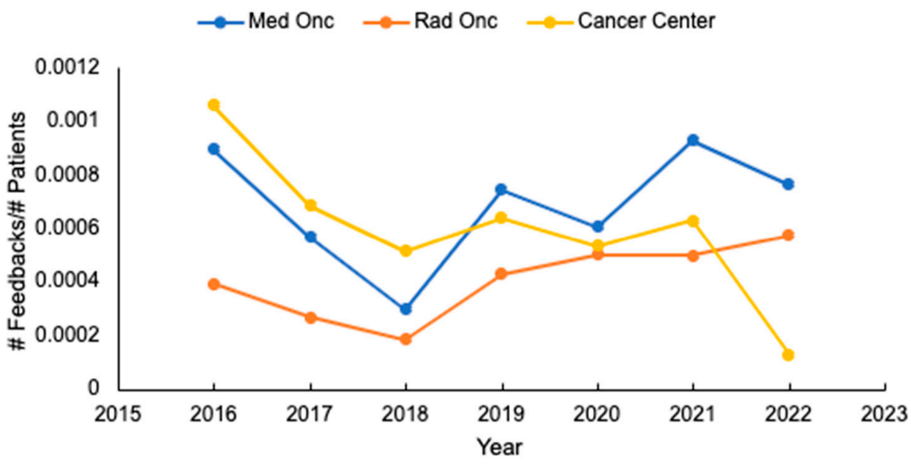

Figure S2. Proportion of feedback per division adjusted for patients seen by year

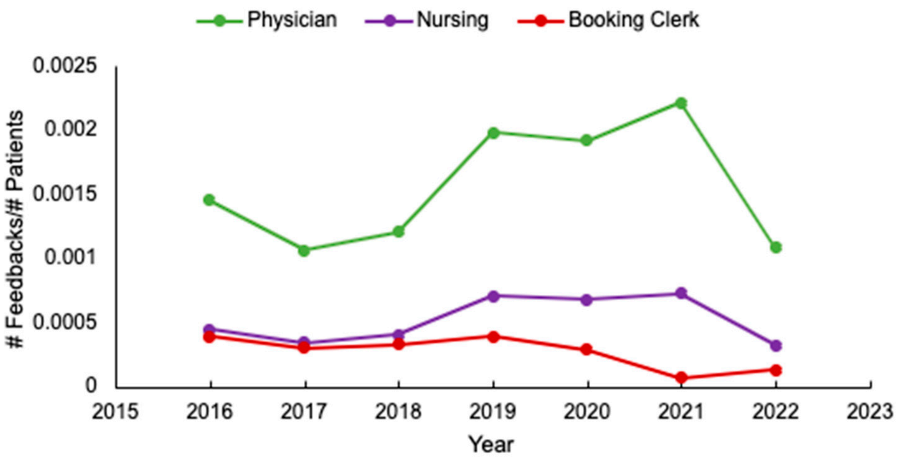

Figure S3 Proportion of feedback per type of staff adjusted for patients seen by year
